# RssAB Signaling Coordinates Early Development of Surface Multicellularity in *Serratia marcescens*

**DOI:** 10.1371/journal.pone.0024154

**Published:** 2011-08-26

**Authors:** Yu-Huan Tsai, Jun-Rong Wei, Chuan-Sheng Lin, Po-Han Chen, Stella Huang, Yu-Ching Lin, Chia-Fong Wei, Chia-Chen Lu, Hsin-Chih Lai

**Affiliations:** 1 Department of Clinical Laboratory Sciences and Medical Biotechnology, National Taiwan University College of Medicine, Taipei, Taiwan, Republic of China; 2 Department of Immunology and Infectious Diseases, Harvard School of Public Health, Boston, Massachusetts, United States of America; 3 Department of Biochemistry and Molecular Biology, Chang Gung University, Kweishan, Taoyuan, Taiwan, Republic of China; 4 Department of Medical Biotechnology and Laboratory Science, and Research Center for Pathogenic Bacteria, Chang Gung University, Kwei-Shan, Tao-Yuan, Taiwan, Republic of China; 5 Department of Respiratory Therapy, Fu Jen Catholic University, Sinjhuang, Taipei, Taiwan, Republic of China; University of Wisconsin-Milwaukee, United States of America

## Abstract

Bacteria can coordinate several multicellular behaviors in response to environmental changes. Among these, swarming and biofilm formation have attracted significant attention for their correlation with bacterial pathogenicity. However, little is known about when and where the signaling occurs to trigger either swarming or biofilm formation. We have previously identified an RssAB two-component system involved in the regulation of swarming motility and biofilm formation in *Serratia marcescens*. Here we monitored the RssAB signaling status within single cells by tracing the location of the translational fusion protein EGFP-RssB following development of swarming or biofilm formation. RssAB signaling is specifically activated before surface migration in swarming development and during the early stage of biofilm formation. The activation results in the release of RssB from its cognate inner membrane sensor kinase, RssA, to the cytoplasm where the downstream gene promoters are located. Such dynamic localization of RssB requires phosphorylation of this regulator. By revealing the temporal activation of RssAB signaling following development of surface multicellular behavior, our findings contribute to an improved understanding of how bacteria coordinate their lifestyle on a surface.

## Introduction

Biofilm formation and swarming are two important bacterial multicellular behaviors on a surface. Bacteria can either resist environmental stress by forming biofilm communities, or rapidly reach a better niche for survival by swarming [1], [2], [3], [4]. In addition to environmental stresses, the resistance to antimicrobials and host-mediated responses are closely correlated with these two behaviors [5], [6], [7], [8], [9], [10]. Therefore, understanding the regulation of biofilm formation and/or swarming is of great importance [11], [12], [13], [14].

When transferred from a liquid medium to a solid surface, bacteria can develop swarming behavior which is divided into at least two stages: (i) the lag period prior to the onset of surface migration, and (ii) active motile migration (or translocation) on surfaces [15], [16], [17]. The transition from the lag period to rapid surface migration is accompanied by cell differentiation and striking morphological changes within the swarming colony [1], [15], [18]. However, the nature of the physiological changes which take place during the swarming lag period and how the environmental challenges induce cell differentiation are poorly understood [12]. Moreover, the investigation of how bacteria coordinate with each other during the swarming lag period is impeded by the fact that no regulatory system has yet been shown to specifically control the duration of this period without affecting migration velocity in swarming development.

In addition to swarming, bacteria can also form biofilms on a surface. Microscopic observations and genetic analyses have revealed a five-stage process in biofilm development including: (i) initial attachment to surfaces, (ii) production of exopolysaccharide resulting in irreversible attachment, (iii) early development of biofilm architecture, (iv) maturation of biofilm architecture, and (v) dispersion of single cells from the mature biofilm [3]. Although several regulatory pathways which regulate swarming motility also control biofilm formation, it is not known at which specific stage(s) during development of both behaviors these regulatory inputs act [19], [20], [21], [22].

When inoculated onto the surface of an LB agar plate (0.8% agar) at 30°C, *S. marcescens* CH-1 exhibits a characteristic swarming phenotype [17], [23]. We have identified and characterized a two-component system (TCS) RssAB, which not only regulates swarming motility but also affects cell-surface attachment and virulence in *S. marcescens* [17], [23], [24]. TCSs consist of sensor kinases and response regulators and play important roles in the adaptation of bacteria to environmental changes [25]. The response regulator generally interacts with its cognate sensor kinase when both proteins are unphosphorylated [25], [26]. Upon sensing signals, the sensor kinase undergoes autophosphorylation, and subsequently transfers a phosphate group to its cognate response regulator. The phophorylated response regulator then usually mediates cellular response through modulation of downstream gene expression [27].

Once signaling is switched ON, the downstream signaling proteins are considered to detach from their cognate partners at the membrane for subsequent signaling events [28], [29]. Examples of such signaling cascades have been reported in the BglF-BglG and EnvZ-OmpR signal transduction systems [30], [31]. In this study, by monitoring the location of EGFP-RssB, we demonstrate that RssAB utilizes a similar mechanism. Our results indicate that in the absence of stimulus, RssB is attached to the cell inner membrane. Upon activation of RssAB, RssB is released into the cytoplasm to bind to its target promoter. By detailed characterization of the phenotype of an *rssBA* deletion mutant and monitoring the activation of RssAB signaling, our results indicate that RssAB signaling delays the initiation of surface migration in swarming development and contributes to maintain normal biofilm architecture during the early stage of biofilm formation.

## Results

### RssAB specifically regulates the duration of the lag period during swarming development

**Figure 1:**
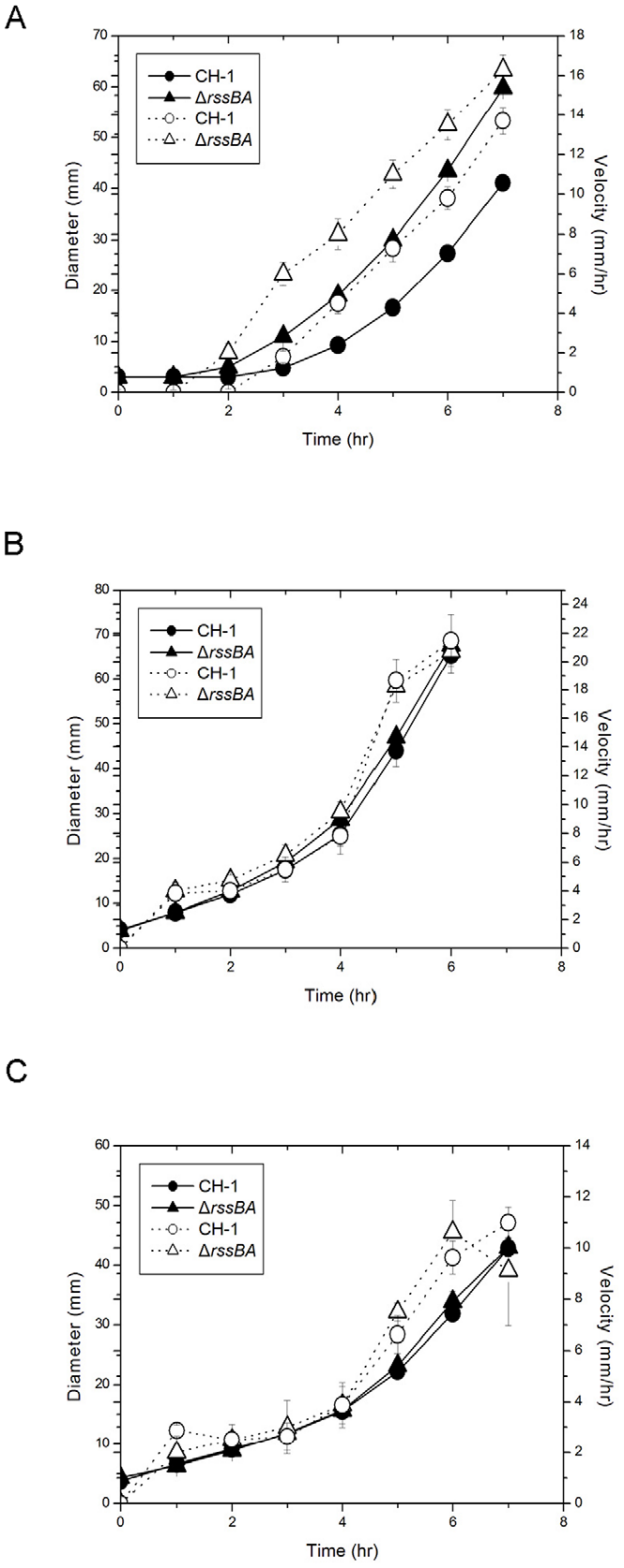
RssAB TCS regulates duration of swarming lag period. Swarming (A) and swimming (C) motility assay of *S. marcescens* CH-1 and isogenic mutant Δ*rssBA* were performed. (B) Swarmer cells harvested from the swarming edge when swarming diameter reached 50 mm were re-inoculated onto another fresh swarming plate at the original density. Diameters (solid line with closed symbols) and velocity (dotted line with open symbols) were shown. Overnight bacterial LB broth culture (1 µl, 10^10^ CFU/ml) was inoculated onto or into the centers of 0.8% (wt/vol) and 0.3% (wt/vol) Eiken agar LB plates at 30°C for swarming and swimming assay, respectively. Results shown were averages of measurements from four independent experiments.

Our previous studies have demonstrated that RssAB negatively regulates swarming motility [17], [23]. Here we further addressed the role of RssAB at different stages during swarming development. Compared with the parental strain *S. marcescens* CH-1, deletion of *rssBA* (Δ*rssBA*) resulted in a reduction in the lag phase by approximately 1 hr without significantly affecting migration velocity at 30°C (Fig. 1A). To verify that RssAB does not affect migration velocity following swarming development, we harvested motile swarmer cells for swarming assay. Both *S. marcescens* CH-1 and Δ*rssBA* swarmer cells migrated immediately following re-inoculation onto fresh LB swarming plates without a lag period, and no significant difference in the migration distance and velocity was observed (Fig. 1B). Swimming motility at 30°C was not affected (Fig. 1C). Briefly, RssAB regulates the duration of the lag period before surface migration in swarming development.

### Deletion of *rssBA* affects biofilm integrity

**Figure 2:**
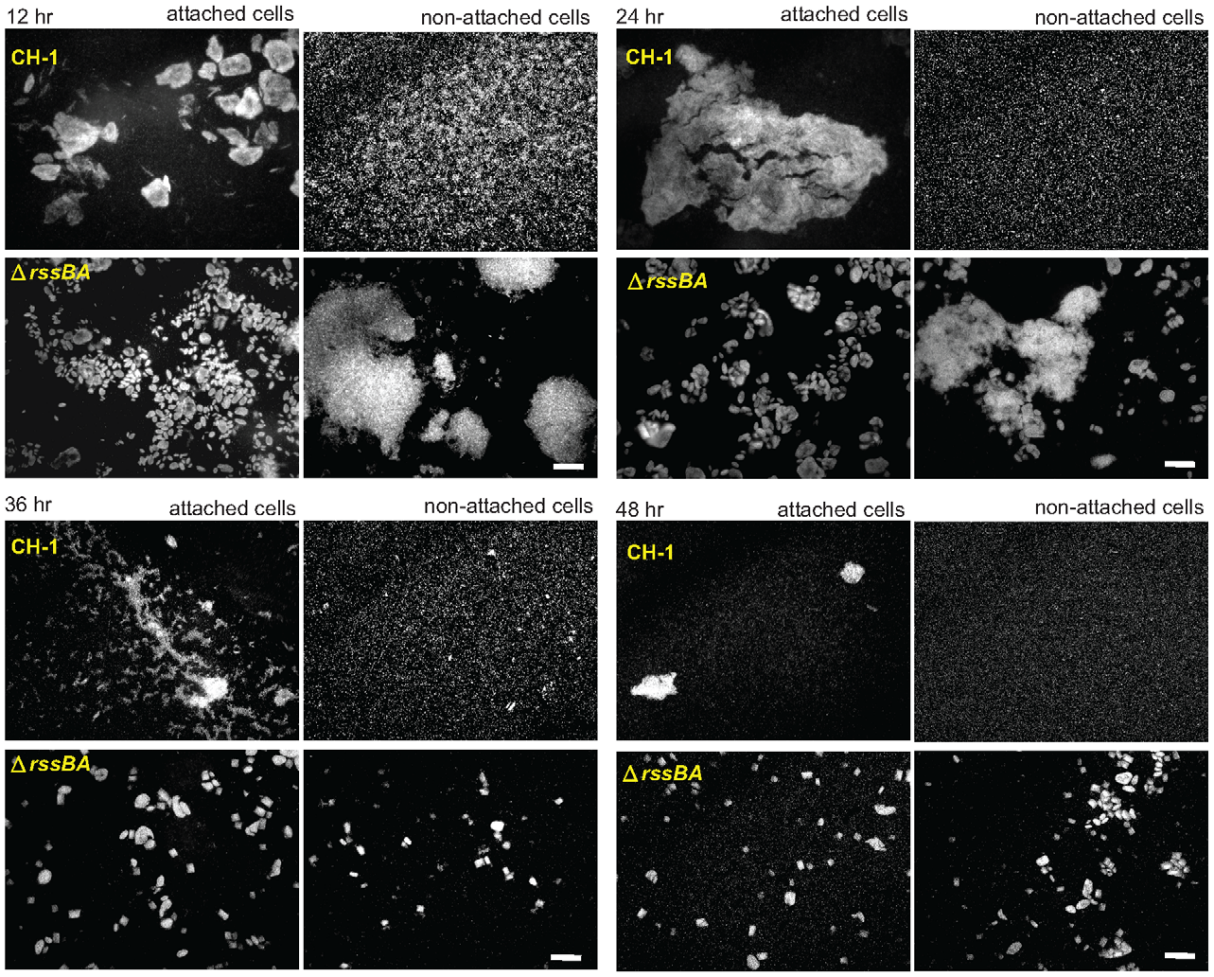
RssAB regulates biofilm structure. Bacteria were cultured in LB medium supplemented with 1% (wt/vol) sucrose and 0.05% (wt/vol) arabinose to overproduce EGFP for observation. Petri dishes with glass coverslips and bacterial suspension were incubated under 50 rpm shaking at 30°C. Biofilm cells attached on glass coverslips or non-attached cells in the broth were observed under fluorescence microscopy at time points indicated. No obvious cell clustering was observed at 6 hr in both attached cells and non-attached cells of *S. marcescens* CH-1 and Δ*rssBA* (data not shown). Scale bar, 50 µm.

We have demonstrated that deletion of *rssBA* results in reduce of cell attachment in biofilm formation [24]. Further detailed examination of both attached and non-attached cell populations of *S. marcescens* CH-1 within the biofilm chamber (petri dishes containing coverslips) with shear force revealed different stages of biofilm development including: aggregation of cells, biofilm maturation, biofilm disassembly and dispersion of single cells (Fig. 2). Compared to *S. marcescens* CH-1 cells which formed mature biofilms after 24 hrs of culture and showed a dispersion of single cells, Δ*rssBA* cells had relatively small cell clusters on surfaces and unusual cell aggregates in non-attached cell population following biofilm development (Fig. 2). The cell aggregates observed in non-attached cell population might come from the sloughs of biofilms since there was no obvious cell aggregate observed under the static culture condition without shear stress, and cell aggregates were also absent when cultured without coverslips which provide adequate surfaces for biofilm formation (data not shown). Taken together, deletion of *rssBA* affects the integrity of the biofilm on surfaces and may trigger biofilm disassembly during the early stage.

### RssB phosphorylation affects its interaction with RssA

**Figure 3:**
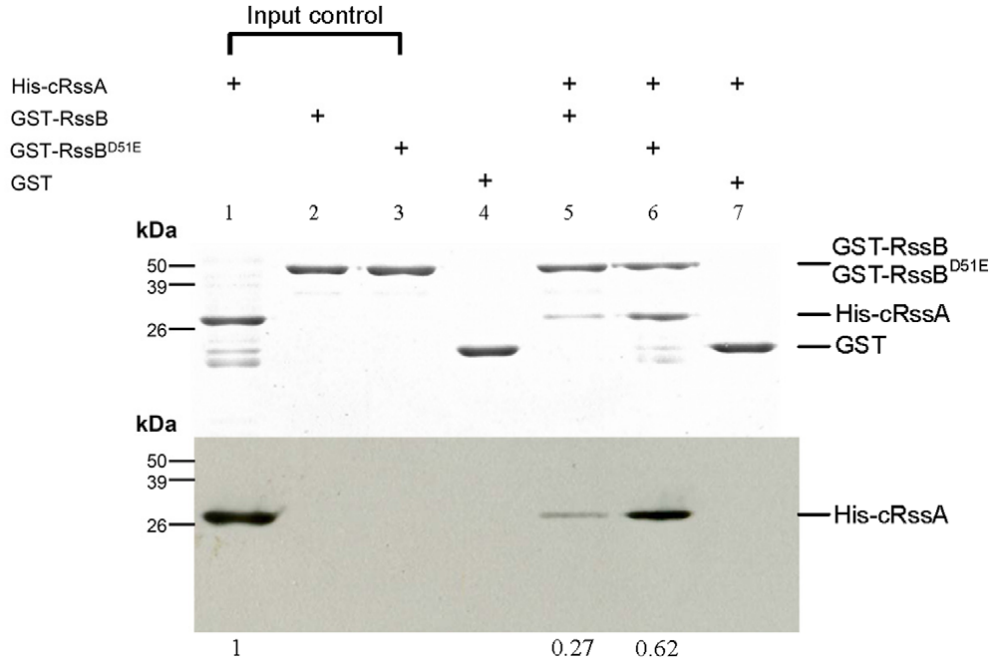
In vitro interaction between RssB and cRssA. GST, GST-RssB and GST-RssB^D51E^ were pretreated with Ac-P, followed by incubation with His-cRssA and glutathione sepharose-4B in interaction buffer. After four times wash with interaction buffer, SDS-PAGE sample buffer was added to each reaction and separated by 12% SDS-PAGE. Proteins were detected by coomassie brilliant blue stain (upper) or anti-hexa-Histidine monoclonal antibody (lower). The molecular weights of GST-RssB and His-cRssA are approximately 50 kDa and 30 kDa respectively. The relative amount of His-cRssA being pulled-down compared to the input one was indicated.

The relationship between RssA-RssB interaction and phosphorylation status of RssB was addressed. We purified GST-tagged RssB (GST-RssB) and its non-phosphorylatable variant GST-RssB^D51E^ respectively and incubated with the poly histidine-tagged cytoplasmic region of RssA (His-cRssA) in the presence of acetyl-phosphate (Ac-P) which acts as a phosphate donor to RssB [23]. We used the mutant protein RssB^D51E^ to mimic the unphosphorylated form of RssB since RssB purified from *E. coli* may be phosphorylated to certain degree and the mutant protein RssB^D51E^ would not be phosphorylated [23]. About 2 times more His-cRssA was pulled-down by GST-RssB^D51E^ (Fig. 3, lane 6) compared to that by GST-RssB (Fig. 3, lane 5), suggesting the unphosphorylated form of RssB (RssB^D51E^) has a higher affinity with cRssA than phosphorylated RssB (RssB∼P). By contrast, no interaction was observed when incubating His-cRssA with GST alone, indicating a specific interaction between cRssA and RssB (Fig. 3, lane 7). This result implied that RssB might be recruited to the cell membrane by RssA in an unphosphorylated form and is released from RssA upon being phosphorylated.

### RssB phosphorylation is required for its dynamic localization in LB broth culture

**Figure 4:**
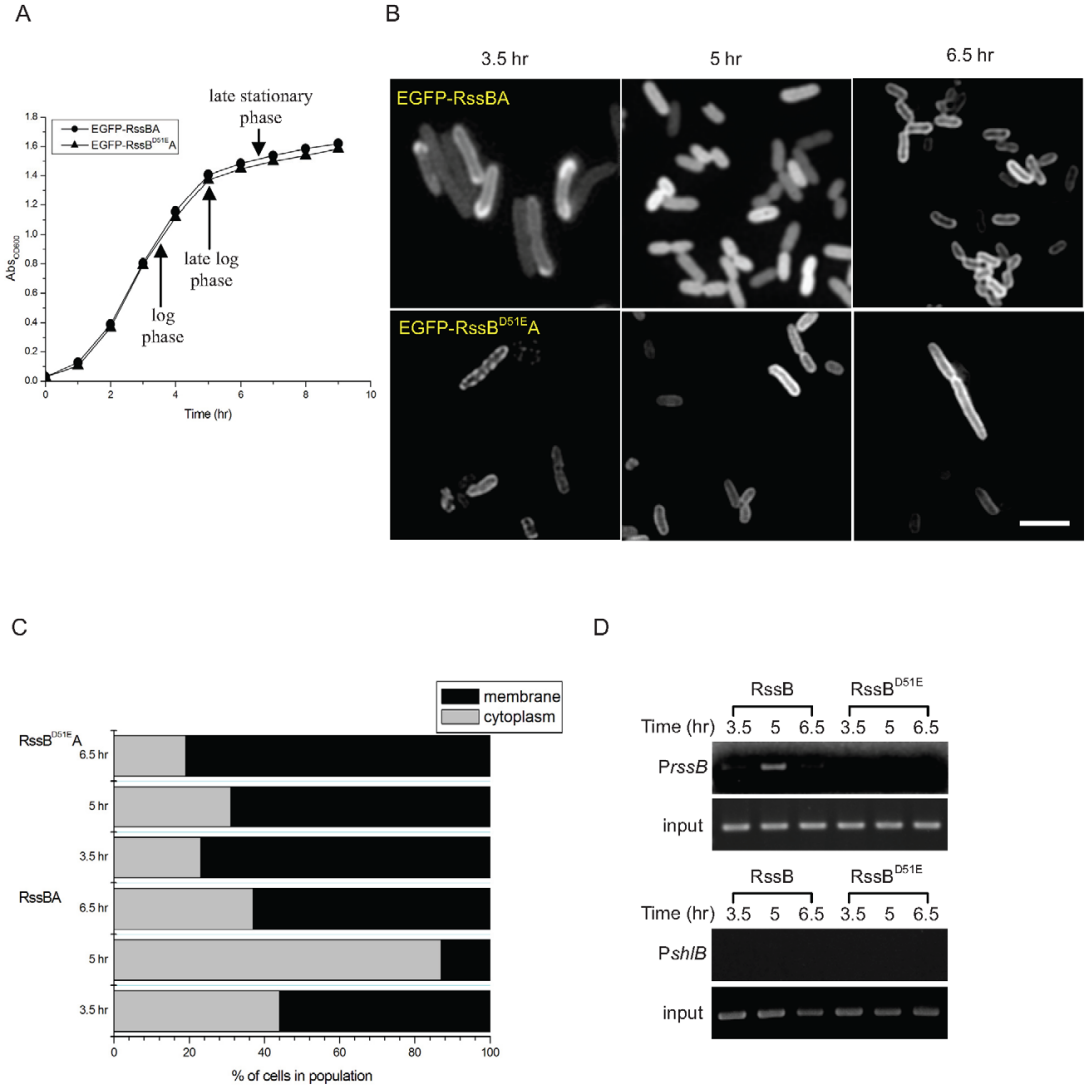
Directly imaging the localization of EGFP-RssB in LB broth culture. (A) The growth dynamic of *S. marcescens* CH-1 harboring pEGFP-RssBA(Sm) or pEGFP-RssB^D51E^A(Sm) was shown. (B) EGFP-RssB was localized at cell membrane in the log (3.5 hr) and the late stationary (6.5 hr) growth phases, and became dispersed in the cytoplasm in the late log phase (5hr) in LB broth culture when comparable amount of RssA was co-expressed. Scale bar, 2 µm (C) The percentage of cells showing EGFP-RssB localizing at the cell membrane or in the cytoplasm. At least 200 cells were counted for each assay condition. For EGFP-RssBA, both EGFP-RssB and RssA were co-expressed under P_BAD_ promoter control and induced by 0.1% (wt/vol) arabinose. EGFP-RssB^D51E^A production was achieved in the same manner. (D) Binding of RssB∼P to its own promoter upon dispersing to the cytoplasm was demonstrated. *In vivo* target DNA binding properties of RssB and RssB^D51E^ were examined by mChIP assay. Captured and total (input) DNA were subjected to PCR using primers PrssBFootF/PrssBFootR and BPshlBF/BPshlBR specific to amplify P*rssB* and P*shlB,* respectively. As RssB∼P cannot directly bind P*shlB in vitro* [23], P*shlB* was used as a negative control.

To monitor the location of RssB which may correspond to the signaling state of RssAB in individual cells, we constructed the recombinant plasmid pEGFP-RssB(Sm) overexpressing EGFP-RssB fusion protein. We have previously demonstrated that RssB down-regulates the expression of its own promoter in the late log phase but not the log phase [23]. We therefore sought to address the localization of EGFP-RssB at 3.5 hr, 5 hr and 6.5 hr which corresponds to the log, late log and late stationary phase, respectively, following the growth in LB broth culture (Fig. 4A). However, no obvious dynamic localization of EGFP-RssB was observed (Fig. S1). If the dynamic localization of EGFP-RssB was due to the direct interaction of EGFP-RssB with RssA, the increased expression of RssA should result in increased localization of EGFP-RssB fluorescence around the periphery of the cell. We thus constructed another plasmid pEGFP-RssBA(Sm), which encodes both untagged RssA and EGFP-RssB fusion protein. The precocious swarming behavior of Δ*rssBA* was successfully complemented by pEGFP-RssBA(Sm), indicating the EGFP-RssB fusion protein functions normally (data not shown). In *S. marcescens* CH-1 cells harboring pEGFP-RssBA(Sm), EGFP-RssB was mostly localized at the cell membrane, forming a bright ring around the periphery of the cell in the log growth phase (3.5 hr), followed by an even distribution within cells in the late log phase (5 hr), and relocalization to the membrane in the late stationary phase (6.5 hr) (Fig. 4B and C). Comparatively, in *S. marcescens* CH-1 cells harboring pEGFP-RssB^D51E^A(Sm), the EGFP-RssB^D51E^ mostly formed a bright ring around the cell periphery in all tested growth phases (Fig. 4B and C, EGFP-RssB^D51E^A). This result suggests that the dispersal of EGFP-RssB into the cytoplasm requires the phosphorylation of this regulator.

We further show that GST-RssB bound to the *rssB* promoter (P*rssB*) in the late log phase (5 hr) stronger than that in the log (3.5 hr) and late stationary phase (6.5 hr), respectively (Fig. 4D). This shows that the increase of RssB binding to its target promoter coincides with its dispersal in the cytoplasm at 5 hr in the late log phase (Fig. 4B and C). By contrast, the non-phosphorylatable GST-RssB^D51E^ did not capture P*rssB* (Fig. 4D), which correlated with the phenomenon that EGFP- RssB^D51E^ mostly located at the cell membrane following LB broth culture when RssA was overexpressed (Fig. 4B and C). Briefly, these results suggest that RssB exists mostly in the unphosphorylated in the log and late stationary phases and binds less effectively to P*rssB*; while in the late log phase, RssB exists mostly in the phosphorylated form, resulting in detachment from the cell membrane, dispersal into the cytoplasm and binding to its target gene promoter.

### Activation of RssAB during the lag period in swarming development

**Figure 5:**
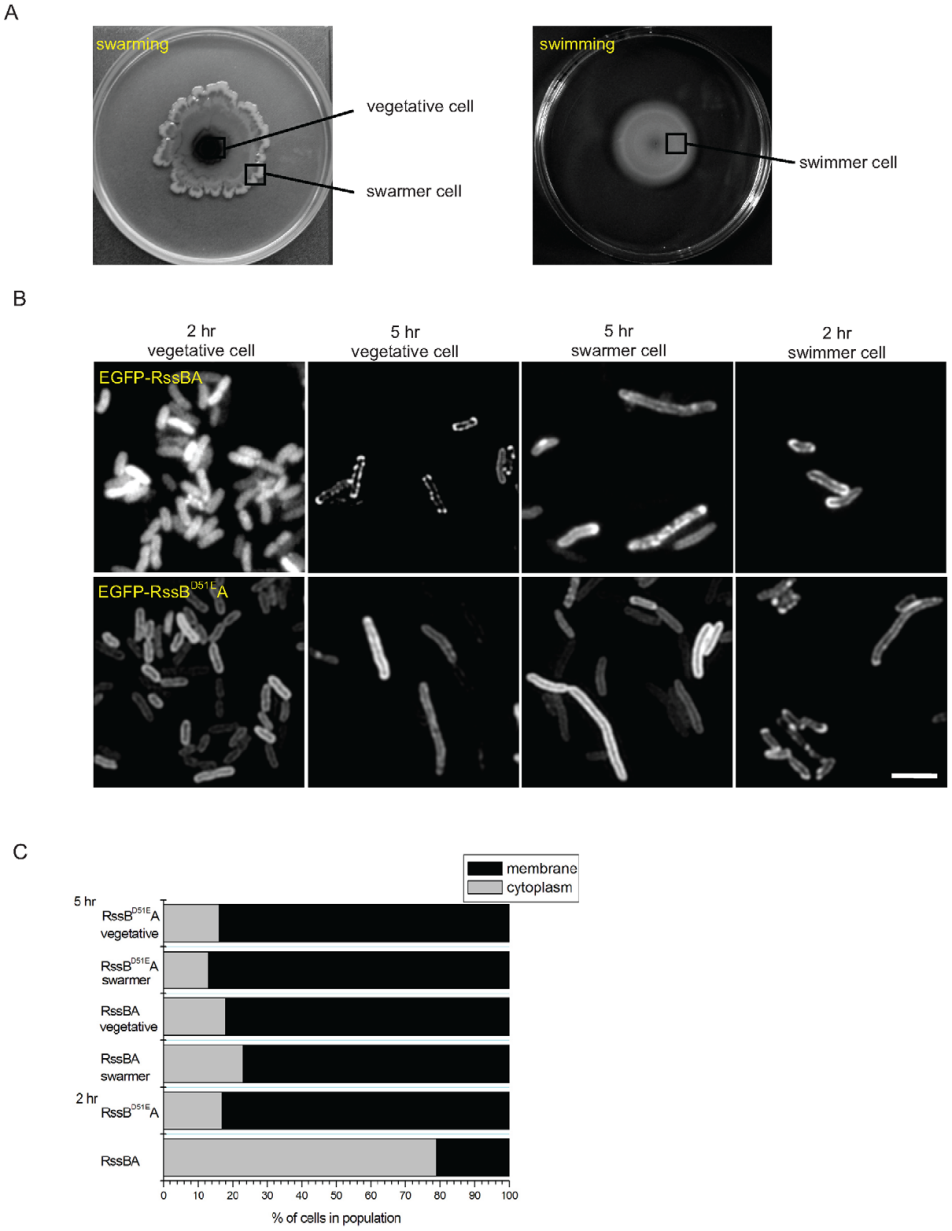
Cytolocalization of EGFP-RssB during swarming development. (A) The patterns of swarming and swimming after 5 hr of inoculation were shown. To image the cytolocalization of EGFP fusion protein in different cell populaiton, cells were taken from the zones of interest as indicated. For the vegetative and swarmer cells, cells were taken from the surface of plates, whereas the swimmer cells were taken from within the agar per se. (B) EGFP-RssB was dispersed in the cytoplasm in central, non-motile cells before initiation of surface migration, and homogenously localized at the periphery of the cell after migration (5 hr vegetative cell and 5 hr swarmer cell) following swarming development. The localization of EGFP-RssB at the cell membrane was also observed in the cells during swimming (2 hr swimmer cell). (C) The percentage of two types of EGFP-RssB localization pattern in cell population cultured on swarming plates was shown. At least 200 cells were counted for each assay condition. For EGFP-RssBA, both EGFP-RssB and RssA were co-expressed under P_BAD_ promoter control and induced by 0.1% (wt/vol) arabinose. EGFP-RssB^D51E^A production was achieved in the same manner. Swarming was performed at 30°C and the bacteria were directly picked by tip and immobilized between a coverslip and a thin slab of 1.5% (wt/vol) agarose for microscopy. Scale bar, 2 µm.

We then addressed the signaling status of RssAB in individual cells following swarming development. Cells were taken from the regions as indicated (Fig. 5A). The vegetative cells were non-motile and the swarmer cells were highly motile on swarming plates under microscopy (data not shown). EGFP-RssB was uniformly distributed in the cytoplasm after 2 hrs of inoculation during the swarming lag period (Fig. 5B and C, 2 hr vegetative cell), but could only be identified at the cell membrane from both vegetative cells and swarmer cells after initiation of surface migration at 5 hr following inoculation (Fig. 5B and C, 5 hr vegetative cell and 5 hr swarmer cell). EGFP-RssB^D51E^ was constitutively located at the cell membrane in *S. marcescens* CH-1 harboring pEGFP-RssB^D51E^A(Sm) following the whole swarming process (Fig. 5B and C, EGFP-RssB^D51E^A). These observations indicate that the RssAB signaling state is ON during the lag period and is OFF once surface migration has begun. In addition, EGFP-RssB was only localized at the cell membrane in swimmer cells, suggesting the signaling state is constitutively OFF during swimming (Fig. 5B, 2 hr swimmer cell).

### RssAB is activated in attached cell aggregates during the early stage of biofilm development

**Figure 6:**
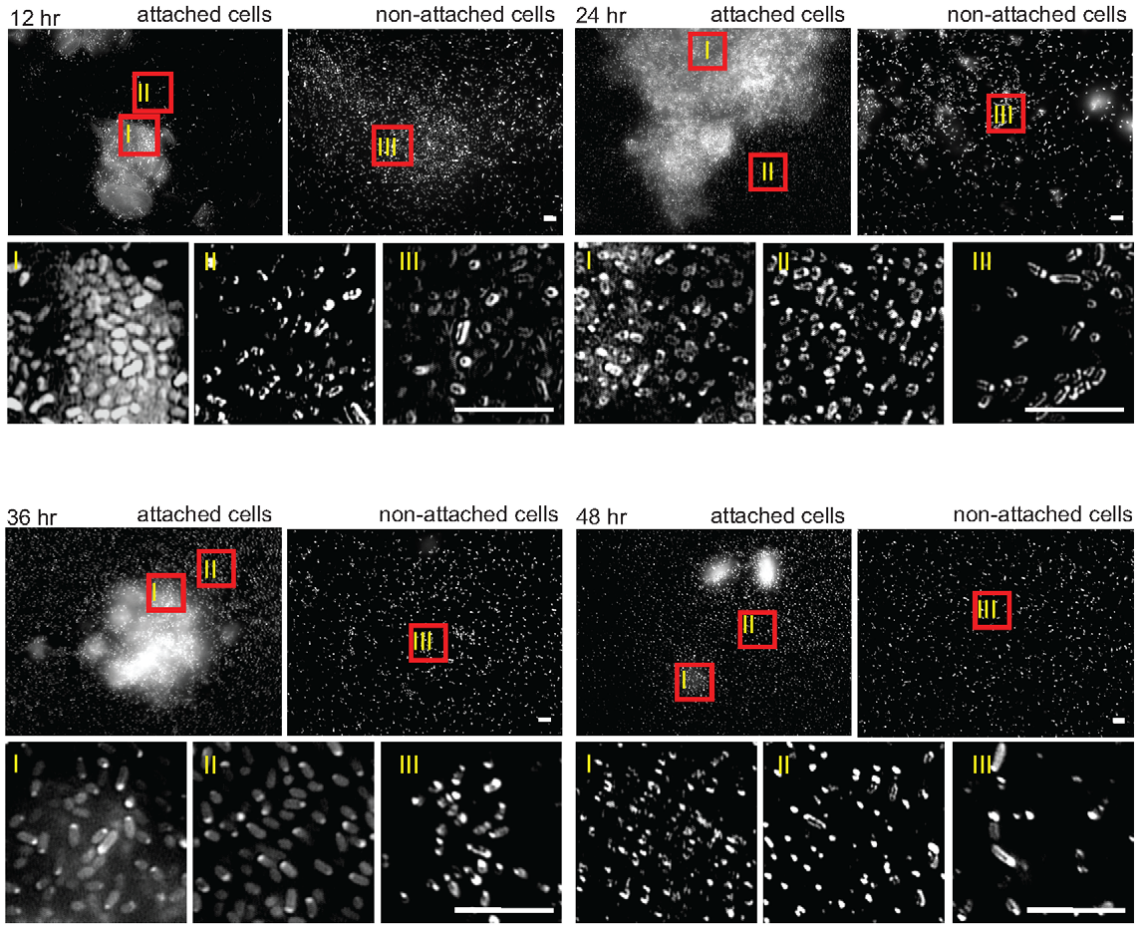
Cytolocalization of EGFP-RssB during biofilm development. Samples were prepared as described in Fig. 2 with cells harboring pEGFP-RssBA(Sm) and observed under 100X objective lens. RssAB signaling status of the cells aggregated on the surface (I), attached on the surface individually (II) and non-attached cells (III) were shown. EGFP-RssB is localized at the cell membrane in both attached cells and non-attached cells following biofilm development except for the 12 hr attached cells in which EGFP-RssB evenly distributed in the cytoplasm when aggregating on the surface. Scale bar, 5 µm.

We also studied the signaling status of RssAB in biofilm development. EGFP-RssB was located at the cell membrane or the cell poles in non-attached cells following biofilm development, suggesting that RssAB was not activated in non-attached cells in these conditions (Fig. 6, non-attached cells). In attached cells, EGFP-RssB was only evenly distributed within cells aggregating on the surface at 12 hr, followed by localization at the cell membrane at 24 hr and clustering at cell poles after 36 hrs (Fig. 6, attached cells). The cluster of EGFP-RssB at cell poles is also observed at 36 hr in LB broth culture when RssA is overexpressed (data not shown). In brief, RssAB signaling only occurs in attached cells during the early stage of biofilm development and is deactivated as the biofilm matures.

## Discussion

As currently no optimal reporter gene is available to real-time monitor RssAB signaling status, we tried to establish a more direct way to address the signaling state of RssAB under different growth conditions. In light of the BglF-BglG and EnvZ-OmpR signal transduction systems in which the dynamic localization of transcription regulators corresponds to the occurrence of signaling and altered transcriptional activity of target genes [30], [31], in this study we developed a fluorescence-based system to directly image the signaling state of RssAB in individual cells.

By imaging the dynamic localization of EGFP-RssB, we reveal the spatiotemporal activation of RssAB signaling in *S. marcescens* in broth culture and also in development of two different surface multicellular behaviors (Fig. S2). RssB is recruited by RssA in a preassembly signaling complex without stimulation. Upon signaling, RssB is phosphorylated and released into the cytoplasm in the late log phase in LB broth culture (Fig. 4 and S2B), and also during the swarming lag period and the early stage of biofilm formation, where RssAB signaling delays surface migration initiation (Fig. 5 and S2C) and affects the structural integrity of biofilms (Fig. 6 and S2D), respectively. When cells enter the stationary phase in broth culture, surface migration begins during swarming, or the biofilm matures, RssB∼P is then dephosphorylated again to form a complex with RssA. In swimmer cells, the signaling is never activated, suggesting RssAB signaling may not involve the regulation of *S. marcescens* swimming behavior (Fig. 5B and S2A). This is consistent with the observation that swimming motility of *S. marcescens* at 30°C was almost not affected in Δ*rssBA* (Fig. 1C). We thus suppose that RssAB may respond to certain degree of cell density to prepare the whole population for responding to the varied environment. As there is no obvious phenotypic change in Δ*rssBA* compared to *S. marcescens* CH-1 growing in the late log phase in broth culture, the potential role of RssAB signaling under this growth condition needs further investigation. Moreover, the evidence that RssAB is only activated at certain stages in development of swarming and biofilm formation suggests that these two behaviors are temporally regulated by RssAB during the whole developmental process.

The EGFP-RssB frequently clusters into spots at the cell membrane or may even only cluster at cell poles when RssAB signaling is in the OFF state (Fig. 5B and 6). The possibility might exist that the polar clustering could be related to the chemotaxis systems, since chemosensory proteins such as CheA and CheY, two TCS proteins regulating chemotaxis often form polar and lateral clusters at the membrane [32]. Whether this phenomenon is dependent on RssA only or is contributed to by other proteins such as chemosensory proteins remains to be addressed.

The fact that RssAB specifically regulates the duration of the swarming lag period and plays no role in regulating migration velocity in swarming development suggests that further study of RssAB in *S. marcescens* is a good model in which to investigate how bacteria regulate the swarming lag period. We also demonstrate that RssAB signaling occurs during the early stage of biofilm formation, which fortifies biofilm architecture following biofilm development. By detection of phosphorylated proteins following biofilm development, several TCSs have been identified to regulate biofilm formation in a stage-specific manner in *Pseudomonas aeruginosa* [33]. However, these TCSs are in a constitutively active state throughout biofilm development once being activated. None of them are temporally activated during the early stage of biofilm formation [33]. Therefore, further identification of downstream genes regulated by RssAB would reveal the cellular response specifically implicated in early development of swarming and biofilm formation.

Our previous study indicated that RssAB down-regulates *flhDC*, the master operon of flagellum biosynthesis [34]. It has been reported that overexpression of *flhDC* results in differentiation of *S. marcescens* MG1 (formerly called *S. liquefaciens* MG1) into swarmer cells to escape the swarming lag period [35]. On the other hand, down-regulation of *flhDC* was suggested to be involved in the irreversible attachment process during the early stage of biofilm formation [36]. We thus suppose that RssAB signaling during the swarming lag period and the early stage of biofilm formation might delay the initiation of swarmer differentiation and ensure irreversible attachment of cells through down-regulating the expression of *flhDC*.

In conclusion, here we characterize the dynamic signaling status of RssAB during the growth in broth culture and the development of multicellular behavior on surfaces. For future studies, it will be interesting to identify the genes regulated by RssAB and the signal inputs of this TCS, to reveal the regulatory network by which bacteria determine whether to act as invasive swarmer cells or be protected in a structured biofilm format while living on a surface.

## Materials and Methods

### Bacterial strains and culture conditions

Both *S. marcescens* CH-1 and its isogenic Δ*rssBA* strain were described in previous studies [17], [24]. *Escherichia coli* DH5α (Invitrogen, U.S.A) was used as a host strain for the maintenance of recombinant DNA plasmids. *E. coli* BL21(DE3)pLysS (Novagen) was used to overexpress recombinant proteins for protein purification. All bacteria used in this study were grown in Luria-Bertani (LB) medium at 30°C or 37°C supplemented with adequate antibiotics when necessary (Table 1). Plasmid construction and primers used in this study were described in supporting information (Text S1 and Table S1).

**Table 1:** Strains and plasmids used in this study.

Strains	Relevant characteristics	Source
*Serratia marcescens*		
CH-1	Clinical isolate, wild type	[17]
Δ*rssBA*	CH-1 Δ*rssBA*::Gm^r^	[24]
*Escherichia coli*		
DH5α	F- *endA1 glnV44 thi*-1 *recA1 relA1 gyrA96 deoR nupG* Φ80*lacZ*_*M15 *_(*lacZYA*-*argF*)U169, *hsdR17*(rK− mK+), λ–	Invitrogen, USA
BL21(DE3)	*E. coli* B F^−^ *ompT hsdS*(r_B_-m_B_-)*dcm* ^+^ Tet^r^ gal (DE3) *endA*	Promega, USA
Plasmids	Description	
pGEX-2T	Expression vector, GST tag	GE Health, USA
pGST-RssB	pGEX-2T-*rssB*-Sm^r^	This study
pGST-RssB^D51E^	pGEX-2T-*rssB* ^D51E^-Sm^r^	This study
pBAD24	pBAD series vector with *pBR* origin	[38]
pBAD24EGFP(Sm)	pBAD24-*egfp*-Sm^r^	This study
pBAD24EGFP(Gm)	pBAD24-*egfp*-Gm^r^	This study
pEGFP-RssB(Sm)	pBAD24-*egfp-rssB*-Sm^r^	This study
pEGFP-RssB^D51E^(Sm)	pBAD24-*egfp*-*rssB* ^D51E^-Sm^r^	This study
pRssA-EGFP(Sm)	pBAD24-*rssA*-*egfp*-Sm^r^	This study
pEGFP-RssBA(Sm)	pBAD24-*egfp*-*rssB*-*rssA*-Sm^r^	This study
pEGFP-RssB^D51E^A(Sm)	pBAD24-*egfp*-*rssB* ^D51E^-*rssA*-Sm^r^	This study
pBluescriptIISK	*lac* promoter, *lacZ* fragment, Amp^r^, CoE1 origin, T7, T3 promoter and primer site, M13 Forward (−20 and −40) and M13 Reverse priming sites, f1 origin	Stratagene, USA

Gm^r^, gentamicin resistant (12 µg/ml); Sm^r^, streptomycin resistant (50 µg/ml); Amp^r^, ampicillin resistant (65 µg/ml).

### Motility assay

Swarming and swimming motility assays were performed on LB medium solidified with 0.8% (wt/vol) or 0.3% (wt/vol) Eiken agar respectively. 1 µl of overnight bacterial LB broth culture (10^9^ CFU/ml) was inoculated onto or into the centers of LB agar plates in swarming or swimming, respectively, and incubated at 30°C.

### Biofilm formation assay

Bacteria were cultured in LB medium supplemented with 1% (wt/vol) sucrose and 0.05% (wt/vol) arabinose to induce the expression of *egfp* for observation. The development of biofilms on glass surface was performed as described by Oh *et al.*, with modification [37]. In this method, we can observe not only the attached cell but also the non-attached cell populations within the biofilm chamber (petri dishes containing coverslips) following biofilm development. Briefly, glass coverslips were pretreated with piranha solution (a mixed solution of 30% H_2_O_2_ and 98% H_2_SO_4_, at a volume fraction of 3∶7) at 60°C for 15 min and rinsed with distilled water. Petri dishes with glass coverslips and bacterial suspension were incubated at 30°C, 50 rpm. For the attached cells, the biofilms on the glass coverslips were removed, coverslips were rinsed with PBS (pH 7.4), and air dried for a few minutes before observation. For the non-attached cells, the cells were removed from the supernatant of the bacterial culture in Petri dishes. Cells were observed under fluorescence microscopy every 12 hrs.

### In vitro protein-protein pull-down assay

Each GST or GST-tagged proteins were phosphorylated by 50 mM acetyl-phosphate at 37°C for 1 hr and added into the mixture containing 100 ng P*rssB* PCR product, 2 µg sheared *S. marcescens* CH-1 chromosome, 25 mM acetyl-phosphate, 1 ml EDTA , 200 µg/ml BSA in 1 ml interaction buffer (20 mM Tris-HCl [pH 7.5], 10 mM MgCl_2_, 100 mM KCl) supplemented with 30 µl 50% glutathione sepharose-4B beads and incubated at 4°C for 1 hr. After centrifugation, the beads were washed with interaction buffer supplemented with 1 mM EDTA, 25 mM acetyl-phosphate and 200 µg/ml BSA three times. The beads were then resuspended in 15 µl interaction buffer for SDS-PAGE analysis and Western blotting.

### Fluorescent microscopy

To express the EGFP and EGFP translational fusion proteins, 0.1% (wt/vol) of sterile arabinose was supplemented when diluting the overnight culture or preparing the swarming plates. In all experiments, 5 µl of bacterial broth culture or cells taken from swarming plates with toothpick were inoculated onto a slide and fixed by 1.5% agarose. Fluorescent microscopy was performed with a Leica DM2500 microscopy under a Leica I3 filter set and observed at 1000x magnification. Images were photographed with SPOT RT3 CCD camera (Diagnostic Instruments, U.S.A) and adjusted by ImageJ (NIH, U.S.A.).

## Supporting Information

Figure S1 **Neither EGFP-RssB nor non-phosphorylatable EGFP-RssB^D51E^ localize at membrane without overexpression of RssA.** Localization of EGFP-RssB and EGFP-RssB^D51E^ was observed in CH-1 cells harboring pEGFP-RssB(Sm) and pEGFP-RssB^D51E^(Sm), respectively. Both EGFP fusion proteins do not localize at the cell membrane in the log (3.5 hr), late log (5hr) and late stationary growth phases (6.5 hr) in LB broth culture. Scale bar, 2 µm.
(PDF)

Figure S2 **Schematic of spatiotemporal activation of RssAB in** ***S. marcescens*** **.** Appearing of green fluorescence (EGFP-RssB) at the cytoplasm and the cell membrane indicates signaling ON or OFF state of RssAB, respectively. (A) In swimming behavior where there is no lag period, cells are freely diffused. RssAB signaling is always at an OFF state. (B) At the late log phase in broth culture, RssAB is activated. The signaling is deactivated after entering the stationary phase. (C) During the lag period preceding surface migration on swarming agar surface, activation of RssAB delays the initiation of surface migration and deactivation of RssAB invokes rapid surface migration. (D) Signaling of RssAB is activated in the aggregates of attached cells during the early stage of biofilm development but deactivated in mature biofilms. In comparison, signaling is always inactivated in non-attached cells following biofilm development.
(PDF)

Text S1 **Supplementary materials and methods.**
(DOC)

Table S1 **Primers used in this study.**
(DOC)
