# Supplementary figures and images for: RssAB Signaling Coordinates Early Development of Surface Multicellularity in Serratia marcescens
Source: PLoS One. 2011 Aug 26;6(8):e24154. doi: 10.1371/journal.pone.0024154 (PMC3162612; doi:10.1371/journal.pone.0024154)

3.5 hr

5 hr

6.5 hr

EGFP-RssB

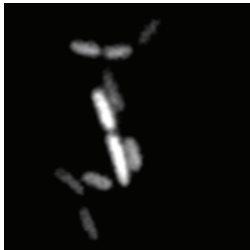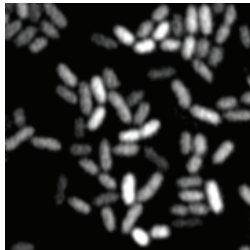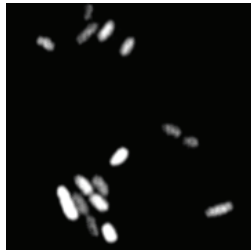

EGFP-RssB<sup>D51E</sup>

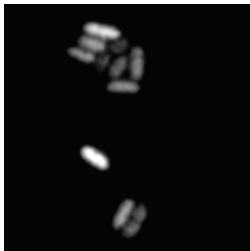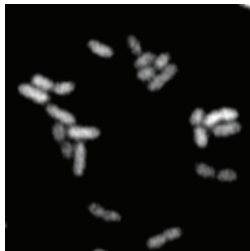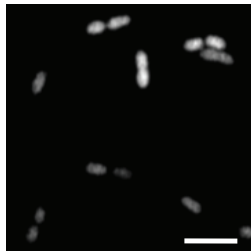

Supplement: Figure S1 — Neither EGFP-RssB nor non-phosphorylatable EGFP-RssBD51E localize at membrane without overexpression of RssA. Localization of EGFP-RssB and EGFP-RssBD51E was observed in CH-1 cells harboring pEGFP-RssB(Sm) and pEGFP-RssBD51E(Sm), respectively. Both EGFP fusion proteins do not localize at the cell membrane in the log (3.5 hr), late log (5hr) and late stationary growth phases (6.5 hr) in LB broth culture. Scale bar, 2 µm. (PDF) [file pone.0024154.s001.pdf]

A

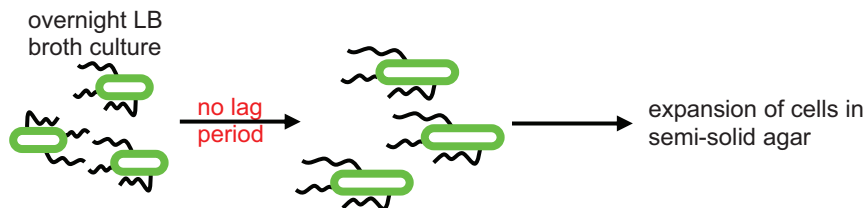

B

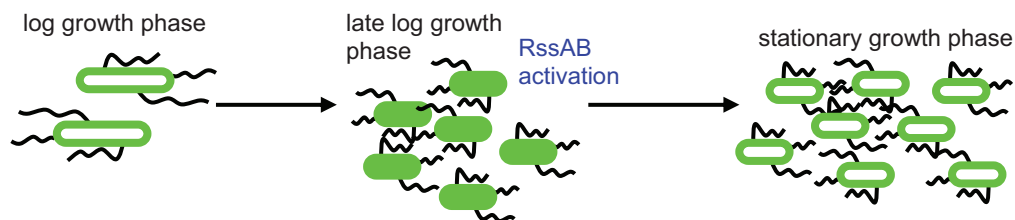

C

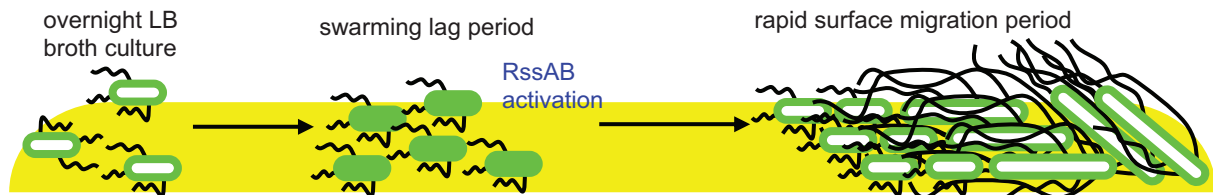

D

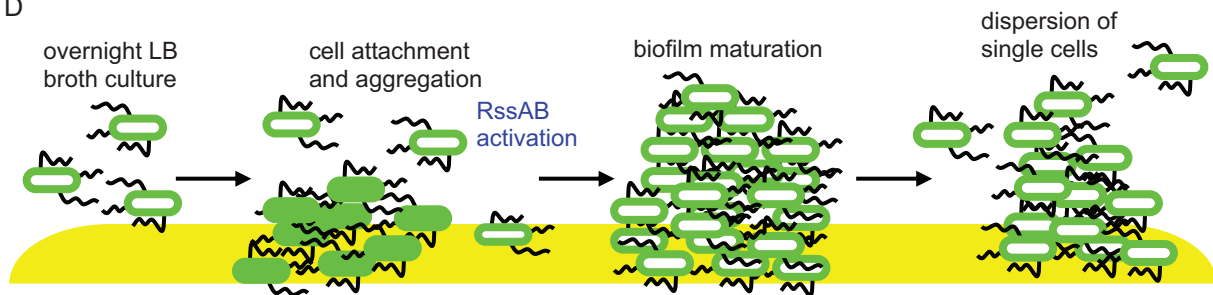

Supplement: Figure S2 — Schematic of spatiotemporal activation of RssAB in S. marcescens . Appearing of green fluorescence (EGFP-RssB) at the cytoplasm and the cell membrane indicates signaling ON or OFF state of RssAB, respectively. (A) In swimming behavior where there is no lag period, cells are freely diffused. RssAB signaling is always at an OFF state. (B) At the late log phase in broth culture, RssAB is activated. The signaling is deactivated after entering the stationary phase. (C) During the lag period preceding surface migration on swarming agar surface, activation of RssAB delays the initiation of surface migration and deactivation of RssAB invokes rapid surface migration. (D) Signaling of RssAB is activated in the aggregates of attached cells during the early stage of biofilm development but deactivated in mature biofilms. In comparison, signaling is always inactivated in non-attached cells following biofilm development. (PDF) [file pone.0024154.s002.pdf]
