# Supplementary material for: RssAB Signaling Coordinates Early Development of Surface Multicellularity in Serratia marcescens
Source: PLoS One. 2011 Aug 26;6(8):e24154. doi: 10.1371/journal.pone.0024154 (PMC3162612; doi:10.1371/journal.pone.0024154)
Supplement: Text S1 — Supplementary materials and methods. (DOC) [file pone.0024154.s003.doc]

**Supporting Information**

**Supplementary Materials and Methods**

*Plasmid construction*

Plasmid pGST-RssB and pGST-RssB(D51E) were created by amplifying the complete *rssB* gene from *S. marcescens* CH-1 chromosomal DNA and pET28RssB(D51E) [1] respectively, using the primer pair BrssBF/ErssBR, and then cloned into the *Bam*HI-*Eco*RI sites of pGEX-2T. In addition, Ω cassette (Sm^r^) from pHP45Ω digested by *Eco*RI was cloned into the *Eco*RI sites of pGST-RssB and pGST-RssB^D51E^.

For the EGFP-RssB localization assay, a PCR product containing the EGFP was firstly amplified from plasmid pEGFP-CIII (BD Biosciences Clontech) using the primer pair SmaEGFPF/XbaEGFPRstop, digested with *Sma*I and *Xba*I, and ligated into the *Sma*I and *Xba*I sites of pBAD24 [2] to form pBAD24EGFP. pBAD24EGFP(Sm) was constructed by introducing an Ω cassette (Sm^r^) from pHP45Ω digested by *Hin*dIII into the *Hin*dIII site of pBAD24EGFP. To construct an N-terminal translational fusion of EGFP to RssB, *egfp* without stop codon was amplified by the primer pair SmaEGFPF/XbaEGFPR and cloned into the *Sma*I-*Xba*I site of pBAD24EGFP(N)(Sm). *rssB* and *rssB*^D51E^ were amplified by primer pair XbarssBF/RssBR from *S. marcescens* CH-1 chromosomal DNA and pET28RssB(D51E) respectively and cloned into *Xba*I-*Pst*I site of pBAD24EGFP(N)(Sm) to form pEGFP-RssB(Sm) and pEGFP-RssB^D51E^(Sm). To construct C-terminal translational fusion of EGFP to RssA, *rssA* was amplified by primer pair ErssAF/SmarssAR and cloned into the *Eco*RI-*Sma*I site of pBAD24EGFP(Sm) to form pRssA-EGFP(Sm). To construct pEGFP-RssBA(Sm) which expresses EGFP-RssB and RssA in one vector, primer pair ErssAF/SmarssAR was used to amplify *rssA* and digested by *Pst*I and *Sma*I; EGFP-RssB was obtained by digestion of pEGFP-RssB(Sm) by *Eco*RI and *Pst*I, and these two fragments were cloned into the *Eco*RI-*Sma*I site of pBAD24EGFP(Sm) to form pEGFP-RssBA(Sm). pEGFP-RssB^D51E^A(Sm) was constructed by the same way otherwise EGFP-RssB^D51E^ was obtained by digestion of pEGFP-RssB^D51E^(Sm) by *Eco*RI and *Pst*I.

*Modified chromatin immunoprecipitation assay*

The modified chromatin immunoprecipitation assay described by Soo and Lai [3] was used with modifications. In all experiments, *in vivo* cross-linking of bacterial nucleoprotein was initiated by the addition of formaldehyde (final concentration of 1%) to cultures. After 10 min, cross-linking was quenched by the addition of glycine (final concentration of 0.3 M). Cultures of 10 ml were collected by centrifugation and washed three times with 20 ml of phosphate buffered saline, resuspended in 1 ml of lysis buffer (10 mM Tris-HCl [pH 8.0], 10 mM EDTA, 20% sucrose) and incubated in 37°C for 30 min. Following lysis, 4 ml of IP buffer (50 mM Tris-HCl [pH 8.0], 150mM NaCl, 0.1% NP-40, 0.1% sodium chloride, 0.5% Triton X-100, 1 μg/ml Leupeptin, 1 μg/ml pepstatin A) was added. Cell extracts were then sonicated to obtain DNA fragments with an average size of 500-1000 bp. Cell debris was removed by centrifugation and 200 μL of the supernatant was retained for use as the input sample in the experiments. For pull-down of GST-RssB cross-linked DNA, 1ml of the supernatant was incubated with 30 μL 50% glutathione sepharose-4B beads (Amersham, UK) at 4°C for 30 min. GST-RssB cross-linked DNA was then pulled down with the glutathione-sepharose 4B beads by centrifugation. The beads were washed twice with IP buffer, three times with LiCl detergent solution (10 mM of Tris, pH 8.0, 250 mM 5 of LiCl, 1 mM of EDTA, 0.5% NP-40, and 0.5% sodium deoxycholate) and twice with TE buffer (50 mM Tris-HCl [pH 7.5], 1 mM EDTA). The beads were then resuspended in a 400 μL of TE buffer and supplemented with 1% SDS. This was followed by incubation at 65°C for 16 hr to reverse the cross-links. DNA samples were purified using phenol extraction, precipitated with isopropanol, resuspended in deionized water and a PCR used to analyze immunoprecipitated DNA.

*Purification of recombinant proteins*

Purification of His-tagged cRssA had been described by Wei and Lai [1]. To over-synthesize GST-RssB, *E. coli* strain BL21(DE3)pLysS containing pGST-RssB or pGST-RssB^D51E^ was grown in 500 ml of LB broth supplemented with 50 µg/ml of ampicilin at 37°C. When OD_600nm_ reached 0.5-0.6, IPTG at a final concentration of 0.2 mM was added, and incubation was continued overnight at 12°C. The GST-tagged RssB were purified as followed. *E. coli* cells were resuspended in PBS (Phosphate based saline) containing 0.1% NP-40, 1 μg/ml leupeptin and 1μg/ml pepstatin A. After incubation for 30 min on ice, bacteria were broken by freezing and thawing, followed by sonication (Misonix, U.S.A). After centrifugation at 12,000× g for 15 min at 4°C, the spent supernatant was filtered and applied to a glutathione sepharose-4B beads (Amersham, UK) before washing three times with PBS. The protein was eluted with 50 mM Tris pH 8.0 containing 10 mM glutathione. The purity of eluted protein was judged to be above 90% based on a coomassie brilliant blue staining assessment. Protein concentrations were determined by measuring absorbance at 595 nm using the Bradford protein assay (Bio-Rad, U.S.A).

**Supplementary References**

1. Wei JR, Tsai YH, Soo PC, Horng YT, Hsieh SC, et al. (2005) Biochemical characterization of RssA-RssB, a two-component signal transduction system regulating swarming behavior in *Serratia marcescens*. J Bacteriol 187: 5683-5690.

2. Guzman LM, Belin D, Carson MJ, Beckwith J (1995) Tight regulation, modulation, and high-level expression by vectors containing the arabinose P_BAD_ promoter. J Bacteriol 177: 4121-4130.

3. Soo PC, Horng YT, Wei JR, Shu JC, Lu CC, et al. (2008) Regulation of swarming motility and *flhDC_(Sm)_* expression by RssAB signaling in *Serratia marcescens*. J Bacteriol 190: 2496-2504.
