# Supplementary material for: RssAB Signaling Coordinates Early Development of Surface Multicellularity in Serratia marcescens
Source: PLoS One. 2011 Aug 26;6(8):e24154. doi: 10.1371/journal.pone.0024154 (PMC3162612; doi:10.1371/journal.pone.0024154)
Supplement: Table S1 — Primers used in this study. (DOC) [file pone.0024154.s004.doc]

Table S1. Primers used in this study

| Primer | Sequence (5’ → 3’) |
| --- | --- |
| BPshlBF | CGGGATCCATTTCGTATTTCCCACTGGTCGG |
| BPshlBR | CGGGATCCGGAAAGGGCCGTGCTGACCAACA |
| SalBKOF | GTCGACCGCCTGATGTACCCGCTGCAAAG |
| HinBKOR | AAGCTTGCAGGTCGTCTTCCACCAACAATAT |
| HinKOF | AAGCTTGAACCACGACCATGATTGCCCCT |
| SalKOR | GTCGACCCTGCGCCAGATAGAAGTCTA |
| SmaEGFPF | TCCCCCGGGATGGTGAGCAAGGGCGAGGAGCTG |
| XbaEGFPR | GCTCTAGAGGGTACTTGTACAGCTCGTCCATGCC |
| XbaEGFPRstop | GCTCTAGAGGTTACTTGTACAGCTCGTCCATGCC |
| HindpBAD24SDF | CCCAAGCTTAGGAGGAATTCACCATGGTAGCCG |
| BProrssBF | CGGGATCCGCCTGATGTACCCGCTGCAAAG |
| HinBKOR | AAGCTTGCAGGTCGTCTTCCACCAACAATAT |
| BrssBF | CGGGATCCATGAACATATTGTTGGTGGAAGACGAC |
| ErssBR | CGGAATTCTACTCTTTCTTCAGCAAATAGCCAAT |
| XbarssBF | GCTCTAGAGATGAACATATTGTTGGTGGAAGACG |
| RssBR | AGAATATTGGCGATGCCTGCAGCG |
| ErssAF | CGGAATTCACCGTACGCGGCATTGGCTATTTG |
| SmarssAR | TCCCCCGGGTCGTGGTTCACTCTCAGTCTG |
| SalrrnBTF | CGCGTCGACGGCTGTTTTGGCGGATGAGAGAAG |

Restriction enzyme sites added to primers are underlined.
